# Cutaneous Metastases in Ovarian Cancer

**DOI:** 10.3390/cancers11091292

**Published:** 2019-09-02

**Authors:** Isao Otsuka

**Affiliations:** Department of Obstetrics and Gynecology, Kameda Medical Center, Kamogawa 296-8602, Chiba, Japan; otsuka.isao@kameda.jp

**Keywords:** ovarian cancer, skin metastasis, radiation therapy, immune checkpoint blockade

## Abstract

Skin metastases in ovarian cancer are uncommon, but their incidence may be increasing due to improved survival rates. Skin metastases can be divided into umbilical metastases, which are known as Sister Joseph nodules (SJNs) and are associated with peritoneal metastasis, and non-SJN skin metastases, which usually develop within surgical scars and in the vicinity of superficial lymphadenopathy. As most skin metastases develop after specific conditions, recognition of preceding metastatic diseases and prior treatments is necessary for early diagnosis of skin lesions. The prognosis of skin metastases in ovarian cancer varies widely since they are heterogeneous in the site of lesion and the time of appearance. Patients with SJNs at initial diagnosis and patients with surgical scar recurrences without concomitant metastases may have prolonged survival with a combination of surgery and chemotherapy. In patients who developed skin recurrences as a late manifestation, symptoms should be treated with external beam radiotherapy and immune response modifiers. Immune checkpoint blockade can enhance anti-tumor immunity and induce durable clinical responses in multiple tumor types, including advanced chemoresistant ovarian cancer. With the use of radiation therapy, which enhances the systemic anti-tumor immune response, immune checkpoint blockade may be a promising therapeutic strategy for distant metastasis, including skin metastasis.

## 1. Introduction

Ovarian cancer is the most lethal gynecological malignancy, since cancer metastasis develops in the majority of patients. Metastatic spread from the primary tumor has often already developed at the time of diagnosis or occurs during the course of the disease. Ovarian cancer spreads directly to the peritoneal cavity by the exfoliation of malignant cells from the tumor surface and also metastasizes via lymphatic channels primarily to the pelvic and para-aortic nodes, and less frequently to inguinal and supraclavicular nodes. In addition, ovarian cancer metastasizes to distant sites through the hematogenous route. The most common sites of distant metastases are the pleura, liver, lung, and lymph nodes [1].

Skin metastases from ovarian cancer are uncommon. Skin metastases of cancers are usually a late manifestation, but in ovarian cancer they are often the first sign [2]. Skin metastases in ovarian cancer include several patterns that differ by site of metastasis and time of appearance. As most ovarian cancers are sensitive to chemotherapy, patients with skin metastasis that develop before initial treatment, almost all skin metastases of that type develop in the umbilicus, respond to the first line chemotherapy consisting of platinum and taxane. Hence, prognosis of patients with ovarian cancer with skin metastasis is not universally poor, although their prognoses have been stated to be poor in previous studies [3,4,5,6,7,8,9,10]. The development of effective chemotherapy and molecular targeted therapy for ovarian cancer has prolonged patient survival and allowed metastases in rare distant sites to implant, grow, and become a clinically evident disease [11].

Manifestations of skin metastases in ovarian cancer vary widely. Thus, a comprehensive understanding of skin metastases is necessary for timely diagnosis, optimizing clinical management and treatment planning, and accurate prediction of prognosis. In this review, possible mechanisms, diagnosis of, and treatment of skin metastasis in ovarian cancer—which includes fallopian tube and primary peritoneal cancers—are discussed.

## 2. Epidemiology

Skin metastases occur in 0.9% to 5.8% of patients with ovarian cancer [1,2,7,12]. The incidence of skin metastases may be increasing in recent years due to improved survival. Ovarian cancers were the primary tumors in 3.3% to 4% of women with skin metastasis [13,14]. The most common primary tumors developing skin metastasis are breast and ovary in women, after exclusion of melanoma, leukemia, and lymphoma [14,15]. In breast cancer, the majority of skin involvement develops by direct extension of underlying primary tumor mass. In contrast, skin metastases from ovarian cancer develop by tumor metastasis. With the exclusion of melanomas, hematologic malignancies, direct tumor extension, and local recurrences, ovarian cancer represented 10% of skin metastases in women [16].

## 3. Patterns

**Figure 1:**
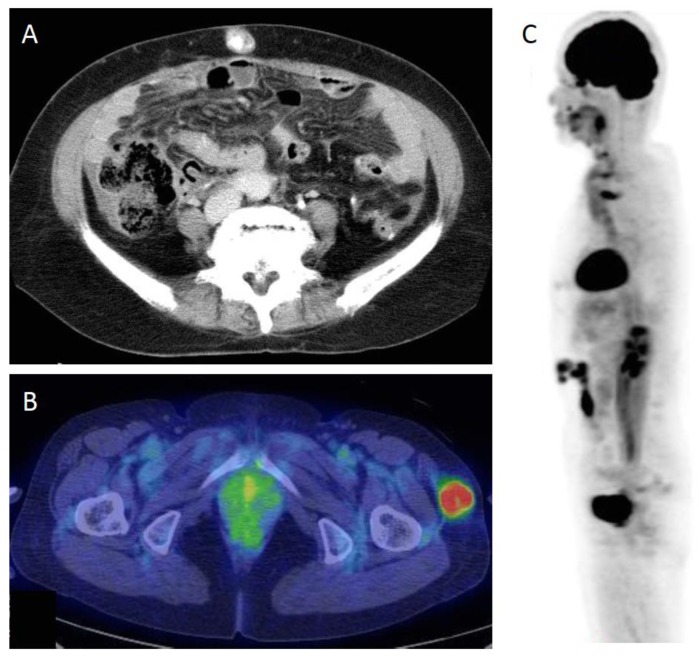
(**A**) CT scan of umbilical metastasis, a Sister Joseph nodule (SJN). (**B**) PET/CT scan of a non-SJN metastasis. (**C**) ^18^F-fluorodeoxyglucose (FDG)-PET image of skin recurrence in surgical scar. FDG uptake is seen in the abdominal surgical scar.

Based on the site of the lesion, skin metastases are classified as metastatic umbilical tumors, which are known as Sister Joseph nodules (SJNs), and non-SJN skin metastases (Figure 1) (Tables S1–S4) [17,18,19,20,21,22,23,24,25,26,27,28,29,30,31,32,33,34,35,36,37,38,39,40,41,42,43,44,45,46,47,48,49,50,51,52,53,54,55,56,57,58]. Skin metastases can further be divided according to the time of appearance: Skin metastases at initial diagnosis and skin recurrences.

### 3.1. Sister Joseph Nodules

Many cases of ovarian cancer with SJNs have been reported in the literature (Tables S1 and S3). SJN refers to a metastatic cancer of the umbilicus and is named after Sister Joseph, a nurse who frequently assisted Dr. William Mayo at St. Mary’s Hospital in Rochester, Minnesota, USA [59]. Although SJNs are considered a late manifestation of a malignant process and representative of an advanced stage of the disease, in ovarian cancer most SJNs are recognized at the time of initial diagnosis. SJN recurrences develop both in patients with concomitant metastases at other sites and those without accompanying metastasis.

### 3.2. Non-SJN Metastasis

Non-SJN skin metastases usually develop in recurrent settings. Many sites were involved in non-SJN skin recurrences: The abdominal wall is the most frequently involved site (Table S3) [1,2,44]. The groin, external genitalia, and chest wall are also involved in skin metastasis. Non-SJN metastases that are recognized at initial diagnosis are rare. Several cases have been reported in the literature, including skin metastases that occur at a site remote from the tumor (Table S2).

## 4. Possible Mechanisms

To understand the mechanisms of skin metastases, the knowledge of preceding conditions, mode of spread of tumor cells, and factors affecting tumor growth is necessary [60]. Skin metastases from ovarian cancer usually develop at the umbilicus, within surgical scars including laparoscopic port sites, and in the vicinity of metastatic superficial lymph nodes (Tables S1–S3).

### 4.1. Preceding Conditions

#### 4.1.1. Intraperitoneal Metastasis

Intraperitoneal metastasis is the most significant risk factor of developing an SJN. Sister Joseph, was the first person to observe that a firm umbilical nodule was often associated with intra-abdominal cancer [59]. A review of the literature showed that 21 of 23 (91%) patients with SJNs at presentation experienced peritoneal dissemination (Table S1). In addition, even though peritoneal dissemination responds completely to chemotherapy, an SJN could develop without accompanying recurrences.

#### 4.1.2. Surgery and Chemotherapy

Skin metastases after surgery can develop at the site of surgical incision. Similarly, many cases of port-site recurrences after laparoscopic surgery have been reported [61,62,63,64,65]. The estimated incidence of port-site recurrences in patients who underwent laparoscopic surgery for malignant disease is approximately 1%–2% [66]. Borderline ovarian tumors, which lack destructive invasion microscopically, can metastasize to port sites after laparoscopic surgery (Table S4).

Adjuvant chemotherapy after surgery reduces the risk of the development of recurrences at surgical incision scars. Non-SJN skin recurrences developing after surgery usually occur in patients who did not receive adjuvant chemotherapy, or patients with a chemo-refractory tumor (Table S3).

In patients with gynecological cancer who underwent laparoscopic surgery, no patients with metastatic ovarian cancer who received chemotherapy developed port-site recurrences [67]. In patients with advanced ovarian cancer who underwent open laparoscopy, which is the separation of the different layers of the abdominal wall through a small incision (minilaparotomy), port-site metastases developed in 17%. However, all port-site metastases disappeared during primary therapy, including chemotherapy, and none of the patients developed a second relapse in one of their port sites [65].

#### 4.1.3. Superficial Lymphadenopathy

Skin metastases can develop in the vicinity of the metastatic superficial nodes. In ovarian cancer, skin metastases in the lower abdomen and the groin develop after inguinal node metastasis, and skin metastases in the chest wall usually develop after axillary node metastasis (Table S3). In addition, SJNs can develop in patients with extensive involvement of the superficial lymph nodes, such as axillary and inguinal nodes.

### 4.2. Mode of Spread

The first step in tumor metastasis is the spreading of tumor cells. In SJNs, contiguous spread and lymphatic flow appear to be important. In non-SJN metastases, direct implantation, extra-nodular extension, and hematogenous metastasis play key roles.

#### 4.2.1. Contiguous Spread

Contiguous spread from the intraperitoneal metastasis appears to be the most common mechanism of SJN occurrence, since the vast majority of patients with metastases to the umbilicus, which is the thinnest part of the abdominal wall, have peritoneal dissemination (Table S1). Thus, SJNs appear to develop by direct invasion from the underlying intraperitoneal metastatic tumors.

#### 4.2.2. Direct Implantation

The direct implantation of viable exfoliated tumor cells is the most likely mechanism through which skin metastasis develops at surgical incisions and laparoscopy port sites, where resected tumor tissues, both invasive and non-invasive, have passed during the procedure. Advanced disease with intraperitoneal metastases and the presence of ascites are risk factors of direct implantation. Port site metastases can occur even at the trocar sites where tumor tissues have not passed. Several mechanisms have been proposed for the development of port-site metastases: Wound contamination and implantation; the multiple effects of pneumoperitoneum; effects of the gases used for insufflation, the “chimney effect;” aerosolization of tumor cells; and surgical technique [66]. Paracenteses in patients with massive ascites can develop skin metastases via direct implantation.

#### 4.2.3. Lymphatic Spread and Extranodular Extension

Skin metastases that develop in the vicinity of superficial lymphadenopathy appear to occur by extranodular extension of the tumor cells from metastatic lymph nodes. Ovarian cancer has three main routes of lymphatic spread: To the para-aortic lymph nodes, to the obturator and pelvic lymph nodes, and to the external iliac and inguinal lymph nodes [68]. Inguinal lymph node metastasis occurs in both patients with pelvic node metastasis by retrograde lymphatic flow and patients without other lymph node metastases. Tumor cells in the inguinal node can extend through the lymph node capsule into the surrounding subcutaneous adipose tissues in the vicinity of the node, and develop skin lesions in the groin, lower abdomen, and external genitalia. The metastatic routes to axillary lymph nodes from ovarian cancer are not known. However, patients with ovarian cancer who develop axillary node metastases have intraperitoneal metastases. Thus, tumor cells may metastasize via subcutaneous lymphatic routes in the upper abdomen to axillary nodes.

Alternatively, SJNs may be caused by spread through lymphatic channels. In a patient who developed an SJN without peritoneal dissemination with inguinal node metastasis, an SJN may result from the alteration of the lymphatic flow by the tumor, which causes the obstruction of lymphatic pathways, shunting the lymphatic flow to the cutaneous lymphatics [69].

#### 4.2.4. Hematogenous Spread

Cancer cells could spread to the skin via hematogenous routes. However, distant metastasis via hematogenous spread is extremely rare in ovarian cancer, whereas circulating tumor cells are found in both advanced- and early-stage disease, 73% and 10%, respectively [70]. An SJN may develop via hematogenous spread. Obliterated umbilical arteries and the urachus could provide pathways for tumor spread [7]. A scalp metastasis has been reported [49].

### 4.3. Factors Affecting Tumor Growth

The second step in tumor metastasis to the skin is the proliferation of tumor cells at the site, which involves wound healing, inflammation, and the presence of adipose tissue.

#### 4.3.1. Wound Healing and Inflammation

After surgery, the colonization and proliferation of tumor cells at the cutaneous tissues use wound healing mechanisms. Many of the molecular mechanisms and signaling pathways that are critical for wound healing have been implicated in cancer cell proliferation [71]. In general, cancer-associated inflammation is characterized as a non-resolving condition: The inflammatory responses during wound healing are hijacked by tumor cells to facilitate tumor growth [72]. Macrophages are crucial drivers of tumor-promoting inflammation [73] and play critical roles in promoting metastatic invasion, proliferation, and the survival of tumor cells through various mechanisms [74]. Tumor-associated macrophages (TAMs) are critical modulators of the tumor environment [75]. TAMs produce growth and survival factors for tumor cells (epidermal growth factor (EGF), fibroblast growth factor (FGF), interleukin (IL)-6, and IL-8) and angiogenic factors (EGF, FGF, vascular endothelial growth factor (VEGF), and platelet-derived growth factor (PDGF)) and suppress the T-cell-dependent antitumor immunity [76].

Angiogenesis, which is the growth of new blood vessels from pre-existing vessels, is essential to cancer progression and wound healing, and is stimulated by inflammation. Angiogenesis is an adaptive method through which cancer cells increase their much needed supplies of oxygen and nutrients when growing, while generating new conduits for distant spreading [77,78]. Neutrophils, macrophages, and endothelial cells in acute wounds secrete VEGF and promote angiogenesis [71]. Following the initiation of angiogenesis, also called the “angiogenic switch,” tumors can more readily expand in size [71]. VEGF also inhibits the functional maturation of dendritic cells [79] and directly triggers regulatory T cell (Treg) proliferation [80], both of which are mechanisms that allow the escape of tumor cells from the host immune system. In addition, Tregs are actively recruited by tumors and suppress both adaptive and innate immune responses [81].

#### 4.3.2. Adipose Tissue

The subcutaneous adipose tissue influences tumor growth, particularly in cases of direct implantation and extranodal extension [82]. In adipose tissues, cancer cells interact with adipocytes and adipocytes de-differentiate into pre-adipocytes or are reprogrammed into cancer-associated adipocytes (CAAs). CAAs secrete adipokines that stimulate the adhesion, migration, and invasion of tumor cells. CAAs release fatty acids through lipolysis, which are then transferred to cancer cells and used for energy production. The abundant availability of lipids from adipocytes in the tumor microenvironment supports tumor progression and uncontrolled growth. The pre-existing inflammation, such as lymphangitis, often associated with lymphedema, may facilitate tumor growth in the adipose tissue. In this environment, adipocytes and inflammatory cells are in an activated state and secrete adipokines and cytokines, which are known to promote tumor development [82]. Chronic inflammation in the skin may alter angiogenesis and/or lymphangiogenesis [83], thus affecting the development of skin metastasis.

## 5. Risk Factors

### 5.1. Tumor Factors

An important tumor factor of skin metastasis in ovarian cancer is tumor histology. Ovarian cancer is a heterogeneous group of diseases that not only behave differently but also develop differently [84]. Some histotypes tend to develop skin metastases, but others do not.

High-grade serous carcinomas are associated with SJNs and surgical incision recurrence, as that type of carcinoma often develops intraperitoneal metastasis. High-grade serous carcinoma is the most common histotype, constituting 70% of ovarian carcinomas [85]. It is thought to originate in the fallopian tube epithelium and spread throughout the peritoneal cavity after exfoliation of tumor cells from the fallopian tube. Also, high-grade serous carcinoma develops skin recurrences after superficial lymphadenopathy; high-grade histology and serous histology are risk factors for lymph node involvement [86]. Low-grade serous carcinoma also develops skin metastases. Low-grade serous carcinoma develops from cystadenoma that arises from epithelial inclusion glands in the ovary, and evidence suggests that epithelial inclusion glands originate in fallopian tubes [87]. Low-grade serous carcinoma is characterized by a young age at diagnosis, relative chemoresistance, and a prolonged survival compared with high-grade serous carcinoma [88]. Late recurrences in the skin, especially at surgical scars, have been reported [4].

Endometrioid carcinoma develops skin metastases. Endometrioid carcinoma is strongly associated with endometriosis, and often originates from endometriotic ovarian cysts. Endometrioid carcinoma tends to develop intraperitoneal metastasis, thus, it is associated with SJNs. Clear cell carcinoma is associated with endometriotic cysts, like endometrioid carcinoma, and develops skin metastases, even in patients with early-stage disease. Clear cell carcinoma is characterized by its chemorefractory nature, thus it develops surgical scar recurrences even though adjuvant chemotherapy is administered [2]. Of note, skin metastases from the mucinous ovarian carcinoma, another type of chemorefractory ovarian carcinoma, have not been reported, whereas in colon carcinoma, an SJN often develops from the mucinous type [89].

Aggressive histotypes, such as undifferentiated carcinoma and neuroendocrine carcinoma, develop distant metastases, especially via hematogenous spread. Borderline tumors also develop skin metastases, but they develop only at port site metastasis. Earlier studies suggested that the invasive properties of the tumor are related to port site metastases [90,91]. However, borderline tumors can metastasize to the subcutaneous tissue. Risk factors include excrescences on ovarian tumors, the presence of ascites, and peritoneal implants (Table S4).

### 5.2. Treatment Factors

The surgical method affects the probability of developing skin metastases after cancer surgery. The probability is higher after laparoscopy than after open surgery [92]. Likewise, the probability is higher at smaller than at larger trocar sites [67]. A study reported that the difference in recurrence rates in 5–10-mm-diameter trocar sites was statistically significant [67]. An explanation for these observations is that tumor cell density, i.e., the number of tumor cells per unit volume, appears to be an important factor for direct implantation.

Port-site metastases after laparoscopic surgery in ovarian cancer are a well-known phenomenon; however, its incidence is low, 1%–2% [66,93], in recently treated patients. Although port-site metastases are a potential complication of laparoscopy even in patients with early-stage disease [64], they usually occur in patients with known metastatic disease and are detected in the setting of synchronous advanced intraabdominal or pelvic metastasis and the progression of carcinomatosis [93]. The presence of ascites and a longer interval between the start of platinum-based chemotherapy and cytoreductive surgery are also risk factors [62] The skin closure type is another risk factor of subcutaneous metastases. The incidence of recurrence at the trocar site has been statistically higher in patients undergoing a laparoscopy in which only the skin was closed at the end of the procedure, than in the patients undergoing a laparoscopy with closure of all layers; i.e., the peritoneum, rectus sheath, and skin [62]. The rate of port-site metastasis after robotic surgery is similar to the rate for laparoscopic procedures [94]. Intraperitoneal chemotherapy may be a risk factor for skin metastases. Skin metastases that develop around the sites of intraperitoneal drug administration have been reported [7,10].

Anti-VEGF antibodies, such as bevacizumab, may influence the development of skin recurrences. Patients with ovarian cancer treated with bevacizumab as secondary therapy after intraperitoneal/intravenous chemotherapy as initial treatment have been reported to be at particularly high risk of extraperitoneal metastases including cutaneous tissue metastasis [95]. Although angiogenesis inhibitors that target the VEGF pathway may restrict tumor growth and metastatic ability [96], they concomitantly elicit tumor adaptation and progression to increase local invasion and distant metastasis occurrence [97]. Acquired resistance is common in VEGF-targeted therapies, and the mechanisms that underlie the modest efficacy of anti-angiogenesis therapies may involve the active recruitment of macrophages to the tumor microenvironment, where they are responsible for the emergence of anti-VEGF therapy resistance [98].

### 5.3. Patient Factors

Obesity is associated with cancer progression and may be related to the development of skin metastases [99]. Obesity is critically related to poor prognosis in cancer, presumably through induction of chronic inflammation [99]. The majority of obese individuals harbor inflamed adipose tissue, which resembles chronically injured tissue, with immune cell infiltration and remodeling [100]. Metabolic syndrome, which includes dyslipidemia and insulin resistance, occurs in the setting of adipose inflammation and promotes tumor growth [100].

Age may play a role in the development of skin metastasis. As we age, the innate immune system becomes dysregulated and is characterized by persistent inflammatory responses that involve multiple immune and non-immune cell types [101]. Age-associated impaired immune function appears to play a pivotal role in the development of distant metastasis. In the elderly, anti-cancer immunity may be compromised because, with aging, (1) the ability of neutrophils and macrophages to phagocytose pathogens decrease and (2) the function of cytotoxic T cells is also compromised [102]. Old age is characterized by low amounts of naïve T cells, exhaustion of potentially tumor-specific memory T cells, and higher amounts of suppressive cells [103]. Older individuals are also more susceptible to inflammatory diseases that promote tumor growth [104].

## 6. Diagnosis

Clinical manifestations and symptoms of skin metastases vary widely, thus diagnosis of skin metastasis is often difficult. The importance of prompt recognition of a skin metastasis is highlighted by potentially being the first clinical sign of a new or recurrent malignancy. Understanding the spectrum of clinical presentations and epidemiology is essential for the timely detection of skin metastases that may allow a prolonged survival with prompt and adequate treatments.

Skin metastasis as the first sign of internal cancer is most commonly seen with cancers of the lung, kidney, and ovary [105]. In a series of patients with skin metastases seen in dermatologic consultation, the underlying cancer had been undiagnosed in 60% of patients with lung cancer, in 53% with renal cancer, and in 40% with ovarian cancer [13]. Skin involvement occurs with internal malignancy in 5% of patients, but it is rarely at the time of diagnosis (1.3%) and even less commonly represents the chief complaint (0.8%) [106].

### 6.1. Sister Joseph Nodule

The presence of SJN can be the first sign of an undiagnosed underlying malignancy or an indication of recurrence in a patient with a known malignancy. SJNs mainly develop in patients with gynecological and gastrointestinal cancers: In a study evaluating 407 patients with SJNs, the most common origins were stomach (23%), ovary (17%), colon and rectum (15%), pancreas (9%), and uterus (6%) [107]. In women, ovarian cancer is the most common origin of SJNs: 42% to 47.7% of women with SJNs had ovarian cancer [17,108]. Of 112 umbilical tumors in one study, 48 (43%) were malignant and 64 (57%) were benign [109]. Another study showed that among umbilical malignancies, 88% originated outside the umbilicus and 12% were primary skin tumors [17]. Differential diagnoses include neoplastic diseases like Paget’s disease, angioma, or squamous cell carcinoma and basal cell carcinoma, and non-malignant diseases, such as umbilical hernia, endometriosis (Villar’s nodule), a hypertrophic scar, pyogenic granuloma, mycosis, psoriasis inversa, and eczema [110].

Patients with an SJN often present with umbilical symptoms, including an umbilical mass, umbilical bleeding, and pruritis. Sometimes, they only have umbilical symptoms without typical ovarian cancer symptoms. The presentation of an SJN can be variable, ranging from a firm and round nodule to a soft and irregular nodule. Its appearance is often misleading because the skin overlying the lesion can be normal or only erythematous [111]. SJNs may be a painful and ulcerated mass. The lesions have also been reported to be variously colored: White, bluish violet, and brownish red [112]. An SJN may grow rapidly or be present for several months before the diagnosis of a malignancy is established [111]. Tumor size varied from 2 × 2 cm^2^ to 10 × 10 cm^2^ [59].

### 6.2. Non-SJN Metastases

Non-SJN skin metastases that develop before the diagnosis of primary cancer are difficult to diagnose when typical cancer-associated symptoms are lacking. However, this type of skin metastasis is extremely rare in ovarian cancer (Table S2). As the vast majority of non-SJN skin metastases develop in recurrent settings, obtaining information on a previous history of ovarian cancer is crucial for the adequate evaluation of skin lesions. Non-SJN skin recurrences usually develop within surgical scars, including sites of trocar incision and drainage tubes. Thus, information on previous surgeries and chemotherapy, particularly the route of drug administration, must be obtained. However, most non-SJN skin recurrences develop during salvage treatments for preexisting metastatic diseases or occur with other concomitant, usually multiple, metastases. Differential diagnoses include cysts, lipomas, fibromas, appendage tumors, cutaneous sarcoidosis, nonmelanoma skin cancer, seborrheic keratosis, inflammatory or infectious processes, and vascular neoplasms [34,113]. Widespread cutaneous metastases on the abdomen, groin, and thigh may be diagnosed as lymphangitis, cellulitis, and herpes zoster [3,44,47].

There are several clinical presentations of non-SJN skin metastases: Isolated cutaneous nodule, multiple cutaneous nodules, cicatricial plaque, and inflammatory metastasis. Most cutaneous metastases arise as nonspecific painless dermal or subcutaneous nodules with an intact, overlying epidermis. They may appear as multiple small papules, sometimes numbering in the hundreds, as large tumors, as sclerotic plaques, or as hemangioma-like nodules [13]. The symptoms of inflammatory metastasis usually start with pitting edema, along with erythematous skin that resembles lymphangitis and cellulitis [3]. In a rare case, an extremely large cauliflower-type tumor grew over a course of four months [4].

### 6.3. Diagnostic Tests

Dermoscopic findings in secondary cutaneous malignancy have not been well described, although dermoscopy is widely used to diagnose skin lesions. In a study of 20 cases of biopsy-proven cutaneous metastases, the most common dermoscopic finding was a vascular pattern, such as serpentine and arborizing vessels, which suggests a role for angiogenesis in their pathogenesis [113]. To accurately diagnose a skin lesion, pathological evaluation is mandatory, especially for nonhealing ulcers, persistent indurated erythema, and unexplained skin nodules, such as new nodules in old scars and nodules in new scars. Fine needle aspiration biopsy is a safe, rapid, reliable, and inexpensive method for the differential diagnosis of lesions [114]. Similarly, an incisional biopsy provides a convenient method of obtaining a tissue sample for histological confirmation of the disease [112]. In asymptomatic patients with skin cancer in whom the primary tumor is unknown, imaging studies, such as computed tomography (CT) scans and positron-emission tomography (PET)/CT, and gastrointestinal evaluation using a gastric fiberscope and colonoscopy, are necessary to identify primary sites. In contrast, skin metastases that develop in patients who already have recurrent disease or patients with persistent disease may not be evaluated pathologically because the diagnosis of skin metastases does not change their management. In these patients, imaging studies, such as CT scans and/or magnetic resonance imaging (MRI), are useful methods. Skin lesions that increase in size during the clinical course and new skin lesions with an abnormal uptake of 18F-fluorodeoxyglucose on PET/CT can be diagnosed as skin metastases [2].

## 7. Prognostic Factors

Prognoses of patients with ovarian cancer who develop skin metastases are not universally poor and are affected by many factors. The sites of skin metastases are an important prognostic factor. Patients with an SJN at initial diagnosis may have a prolonged survival, although almost all SJNs are associated with intraperitoneal metastases. In ovarian cancer, metastatic diseases at presentation respond to initial therapy, including chemotherapy. Most common histotypes of ovarian carcinoma related to SJNs are serous or endometrioid tumors, both of which are chemotherapy-sensitive. In addition to SJN recurrences, skin recurrences within surgical scars may have prolonged patient survival with resection, as they often develop as a solitary lesion without coexisting metastases. In addition, SJNs often develop as a solitary recurrent lesion in patients with serous or endometrioid carcinoma after complete response to initial treatment, including chemotherapy.

The presence of concomitant metastases carries a poor prognosis, particularly in recurrent settings. Skin metastases developing after recurrence in other sites, such as lymph node metastasis and/or peritoneal metastasis, usually occur with other coexisting metastases. Almost all patients with these skin recurrences have already received many cycles of chemotherapy and have become chemotherapy-resistant, thus treatment options are limited.

Skin recurrences in borderline tumors have a favorable prognosis, even though metastatic lesions develop immediately after laparoscopy (Table S4). Eight cases have been reported in the literature, and all patients were salvaged with resection of the lesion with or without chemotherapy. However, an invasive recurrence may develop in patients with borderline tumors [58].

Time to recurrence, i.e., time interval between the initial diagnosis and skin recurrences, may affect survival after skin recurrences [7]. In patients who received chemotherapy as an initial treatment, the chemosensitivity of the recurrent tumor depends on time to recurrence; patients with a long time interval usually have a chemosensitive tumor. Late recurrences, i.e., recurrences that develop five or more years after initial treatment, are rare in ovarian cancer; however, they may not be uncommon in skin recurrences. In general, late recurrences in ovarian cancer are characterized by earlier stages, non-serous histology, and the absence of symptoms at initial diagnosis [115]. However, in skin metastases, late recurrences usually develop in advanced cancer of serous histology. In addition, a low-grade tumor and the absence of *BRCA* mutations appear to be associated with late recurrence [4,116].

The patient’s general condition is also an important prognostic factor. Cancer treatment may often not be performed for the oldest women aged ≥80 years with poor performance status.

## 8. Treatment

Patients with an SJN at initial diagnosis should be treated with the current standard treatment for advanced ovarian carcinoma—a combination of cytoreductive surgery, including SJN resection and adjuvant platinum/taxane chemotherapy, with or without bevacizumab. Patients should undergo primary cytoreductive surgery if optimal debulking appears possible based on findings of imaging studies. Neoadjuvant chemotherapy should be considered when extensive abdominal metastases and ascites are present. In our previous study, three patients received paclitaxel/carboplatin chemotherapy and survived more than 22 months [2]. A review of the studies in which treatment and survival data are provided showed that the median survival of patients with SJN at presentation, who received platinum and/or taxane chemotherapy, is 26 months, which compares favorably with the 25-month survival of patients with stage IV disease who underwent primary debulking surgery [2,117].

Conversely, patient survival with SJN developing as a recurrent disease may not be favorable; it is affected by coexisting recurrent diseases and the time to recurrence. For patients with an SJN recurrence without other concomitant metastases, surgical resection may be a treatment option. Chemotherapy should be provided to patients with coexisting peritoneal metastases that develop after a long-term disease-free interval (DFI).

In patients with a solitary skin recurrence developing within surgical scars, surgical resection appears to be an effective treatment option when no other metastasis coexists. In these patients, a complete resection of the skin lesion is necessary to improve survival, similar to other secondary cytoreductive surgeries. Surgical resection may be an adequate treatment for port-site metastases of borderline tumors. External beam radiotherapy may be effective for a localized chemotherapy-resistant lesion [118,119].

For other types of skin metastases, individualized management is required. Systemic chemotherapy should be administered in patients with non-SJN metastasis at presentation and patients who developed recurrences after long-term DFI. However, the majority of patients with skin metastases are patients with extensive skin metastases or patients with other coexisting metastases, thus in those patients, the treatment aim is to palliate symptoms and to provide better quality of life [8]. Electrocoagulation has been successfully used for local control of pain, hemorrhage, and infection [120]. Mohs’ chemosurgery, i.e., a technique of chemical fixation of a cutaneous tumor using 10% zinc chloride, may be a palliative treatment option [121]. External beam radiotherapy is a feasible and efficient treatment option for extensive skin metastasis with minimal morbidity [118,119]. In a previous report, dramatic remission of skin metastasis treated with chemotherapy and radiation therapy with the dose of 50 Gy was observed [4]. Focal radiation therapy can stimulate a systemic anti-tumor immune response and lead to regression and rejection of non-irradiated, distant tumor lesions (abscopal effect) due to interferon induction and activation of anti-tumor T cells [122].

Immunostimulatory agents may be a treatment of choice for skin metastases. Imiquimod, an immune response modifier, may be effective in skin metastasis [123]. Imiquimod is thought to enhance the immune response against tumors by stimulating dendritic cells and macrophages and by activating inflammatory cytokines and chemokines through toll-like receptors. In addition, it has antiangiogenic properties and can stimulate intrinsic apoptosis [123]. Catumaxomab, a trifunctional bispecific antibody directed against the epithelial cell adhesion molecule and T-cell antigen CD3, is administered intraperitoneally for the treatment of malignant ascites. In a patient with ovarian carcinoma with malignant ascites, a clinical response of skin lesions was observed for intraperitoneal catumaxomab [41]. Intraperitoneal catumaxomab infusion activates natural killer cells and macrophages, in addition to T cells in ascites, and favors CD8+ T cell accumulation in ascites [124].

Immune checkpoint inhibitors, i.e., those targeting the programmed cell death 1, programmed cell death ligand 1, and cytotoxic T lymphocyte antigen 4 pathways, can enhance anti-tumor immunity and induce durable clinical responses in multiple tumor types, including advanced chemoresistant ovarian cancer [125]. Cancers co-opt certain immune checkpoint pathways as a major mechanism of immune resistance, particularly against T cells that are specific for tumor antigens. An immune checkpoint blockade can activate T cells to destroy tumors and may be a potentially curative therapeutic approach.

## 9. Conclusions

Skin metastases have been considered to be a sign of poor prognosis; however, in patients with ovarian cancer, the prognosis of skin metastasis is not always poor. Skin metastases in ovarian cancer usually develop after specific preceding conditions. Adequate treatment should be chosen based on the site of skin metastasis and the presence or absence of concomitant metastases. Surgical resection should be considered for SJNs and surgical scar recurrences when concomitant metastases are absent. With the use of radiation therapy, which enhances the systemic anti-tumor immune response, immune checkpoint blockade may be a promising therapeutic strategy for distant metastases, including skin metastases.

## Supplementary Materials

The following are available online at https://www.mdpi.com/2072-6694/11/9/1292/s1, Table S1: Patients with ovarian carcinoma with Sister Joseph’s nodule at initial diagnosis who received chemotherapy; Table S2: Patients with non-SJN metastasis that developed at initial diagnosis; Table S3: Clinical features of skin recurrences; Table S4: Port-site recurrence of borderline ovarian tumor.
