# Supplementary material for: Cutaneous Metastases in Ovarian Cancer
Source: Cancers (Basel). 2019 Sep 2;11(9):1292. doi: 10.3390/cancers11091292 (PMC6788186; doi:10.3390/cancers11091292)
Supplement: Supplementary file 1 [file cancers-11-01292-s001.pdf]

Supplementary Materials

Cutaneous Metastases in Ovarian Cancer

Isao Otsuka

Table S1. Patients with Sister Joseph's nodule at initial diagnosis who received chemotherapy.

No.	Author (Year)	Age	Histotype	Presenting Symptoms	Metastases Concomitant to Skin Met	Treatment	Survival (months)	Status
1	Brustman (1984) [17]	73	Papillary cystadenoca	Ulcerated, bleeding umbilical mass	Peritoneal D	Surgery, Chemo (CAP-H)	2	DOD
2	Brustman (1984) [17]	59	N/A	NA	Peritoneal D	Surgery, Chemo (CAP-H)	20	DOD
3	Kato (2000) [18]	76	Serous papillary (FTCa)	Umbilical bleeding	Peritoneal D	Surgery, Chemo (CAP)	5	Alive
4	Hashimoto (2000) [19]	81	Serous G2	NA	Peritoneal D	Surgery, Chemo (Carbo/CTX)	7	NED
5	Hashimoto (2000) [19]	47	Serous G1	NA	Peritoneal D	Surgery, Chemo (TC, CTX)	11	NED
6	Yu (2008) [20]	82	Serous	Umbilical mass	Peritoneal D	Surgery, Chemo (TC)	20	Alive
7	Kurt (2009) [21]	49	Mixed type (endometrioid G3 + serous)	Umbilical mass, constipation, abdominal pain	Peritoneal D	Surgery, Chemo (TC)	15	DOD
8	Fukushima (2014) [22]	65	Serous G3	Umbilical mass	Peritoneal D	Surgery, Chemo (TC)	25	AWD
9	Otsuka (2017) [2]	72	Serous, G1	None	Peritoneal D	Surgery, Chemo (TC)	27	DOD
10	Otsuka (2017) [2]	61	Clear cell	None	Peritoneal D	Surgery, Chemo (TC)	22	DOD
11	Otsuka (2017) [2]	70	Serous, G3	None	Peritoneal D	Chemo (Etop, TC)	42	DOD
12	Kirshtein (2006) [23]	54	Poorly diff (FTCa)	(Umbilical hernia)	Peritoneal D (implants)	Surgery, Chemo (Carbo)	13	NED
13	Calista (2002) [24]	65	Adenoca, G3	Umbilical mass	Peritoneal D, LN (retroperitoneal)	Surgery, Chemo (CAP)	6	DOD
14	Matsubara (2004) [25]	59	Serous	Umbilical bleeding	Peritoneal D, LN (pelvic)	Surgery, Chemo (CAP)	8	NED
15	Mizuno (2006) [26]	70	Serous	Umbilical bleeding and itching	Peritoneal D, LN (retroperitoneal)	Surgery, Chemo (PTX)	6	NED
16	Haneda (2001) [27]	74	Serous	Umbilical mass	Peritoneal D, LN (retroperitoneal)	Surgery, Chemo (Carbo/CDDP)	14	DOD
17	Yokota (2014) [28]	70s	Endometrioid, G1	Umbilical bleeding	Peritoneal D, LN (retroperitoneal)	Surgery, Chemo (TC)	17	NED
18	Fukushima (2014) [22]	45	Serous G3 (FTCa)	Umbilical mass, Inguinal mass	Peritoneal D, LN (paraaortic)	Surgery, Chemo (CDDP, PTX)	33	DOD
19	Ohta (2007) [29]	51	Endometrioid, G3	Umbilical mass	Peritoneal D, LN (inguinal)	Surgery, Chemo (TC)	10	NED
20	Kolwijck (2007) [30]	18	Serous	abdominal pain, bleeding and itching umbilicus	Peritoneal D, Pleura, LN (inguinal)	Surgery, Chemo (TC, CAP, TAM, LPD)	28	DOD
21	Cormio (2003) [7]	67	Serous, G3	NA	Peritoneal D, Pleura	Surgery, Chemo (TC)	25	DOD
22	Mimura (2018) [31]	47	Serous, HG	Umbilical pain and bleeding	LN (retroperitoneal, inguinal)	Surgery, Chemo (TC + Bev)	24	NED
23	Haraguchi (2007) [32]	82	Adenoca, G2	Umbilical mass with discharge	None	Surgery, Chemo (TC)	31	AWD

Peritoneal D, peritoneal dissemination; LN, lymph node; NA, not available; FTCa, fallopian tube carcinoma; CAP-H, cyclophosphamide (CTX), Adriamycin, cisplatinum (CDDP), hexamethylmelamine; TC, paclitaxel (PTX)/carboplatin (Carbo); TAM, tamoxifen; LPD, liposomal doxorubicin; Etop, etoposide; DOD, dead of disease, NED, no evidence of disease; AWD, alive with disease

Table S2. Patients with non-SJN metastasis that developed at initial diagnosis.

No.	Author (Year)	Age	Site	Histotype	Symptoms Related to Skin Met	Metastases Concomitant to Skin Met	Treatment	Survival (months)	Status
1	Waraich (2006) [33]	49	Chest wall	Undifferentiated	Chest wall nodules	Peritoneal D Inguinal/para-aortic LN	Surgery (BSO + Om)	<1	NA
2	Antonio (2016) [34]	58	Nose	Undifferentiated	Painful nodule on the nose	Peritoneal D	Palliative Chemo	3	DOD
3	Cowan (1995) [5]	81	Abdomen (lt lower quadrant)	Serous (Perit Ca)	Nodule	Peritoneal D	Surgery Chemo (CP)	12	NED
4	Rieger (1998) [35]	85	Port-site (after laparoscopy of other disease)	Moderately-differentiated Adenoca	Painful nodule at port site	Peritoneal D	None (Exploratory laparotomy)	2	AWD

Peritoneal D, peritoneal dissemination; LN, lymph node; BSO, bilateral salpingo-oophorectomy; Om, Omentectomy; NA, not available; Perit Ca, peritoneal carcinoma; CP, cyclophosphamide/cisplatin; DOD, dead of disease; NED, no evidence of disease; AWD, alive with disease.

Table S3. Clinical features of skin recurrences.

No.	Author (Year)	Age	FIGO Stage at Diagnosis/ Histotype	Previous Therapies	Time to Skin Rec	Preceding Conditions	Site of Skin Rec	Metastases Concomitant with Skin Rec	Treatment	Status/ Survival
SJN Recurrence										
1	Otsuka (2017) [2]	49	IIIC/ Endom G2	Chemo (TC), Surgery	115 mos	Peritoneal D	Umbilicus	None	Surgery	AWD/ 94+ mos
2	Otsuka (2017) [2]	58	IIIC/ Serouo G1	Chemo (TC), Surgery	28 mos	Peritoneal D, Inguinal LN	Umbilicus	Peritoneal D	Chemo (DTX, PLD)	DOD/ 33 mos
3	Otsuka (2017) [2]	45	IIIC/ Serous	Chemo (TC), Surgery	39 mos	Peritoneal D	Umbilicus (1st rec)	Peritoneal D	Chemo (TC, CPT-11)	DOD/ 26 mos
4	Tsai (2006) [36]	72	IIIC/ Serous	Surgery, Chemo (CAP)	8 yrs	Peritoneal D	Umbilicus (1st rec)	None	Resection, Chemo (TC)	NED/ 22 mos
5	Tsai (2006) [36]	44	IIC/ Endom	Surgery, Chemo (CAP)	3 yrs	Pelvic peritoneal D	Umbilicus (1st rec)	None	Resection, Chemo (TC)	NED/ 12 mos
6	Gupta (2014) [9]	56	IA/ Serous	Surgery	2 yrs	None	Umbilicus (1st rec)	None	Resection, Chemo	Alive/ NA
7	Nikolaou (2013) [37]	66	IIA/ Endom	Surgery, Chemo	8 yrs	Pelvic D	Umbilicus	Peritoneal D, liver	Palliative Chemo (PTX, etop)	DOD/ 11 mos
8	Husein-Elahmed (2010) [38]	78	IVB/ low-diff adeno	Surgery, Chemo (cap/oxa)	2 mos	Sigmoid colon, liver	Umbilicus (1st rec)	Sigmoid colon, liver	Chemo (TC)	Alive/ 6 mos
9	Cheng (2017) [10]	67	T3c/ Serous G3	IP chemo (TC), Surgery	2 yrs	Peritoneal D, Puncture (IP chemo)	Umbilicus (1st rec)	None	Chemo	DOD/ 13 mos
Non-SJN Recurrence within surgical scars										
10	Otsuka (2017) [2]	48	IC1/ Clear cell	Surgery, Chemo (MMC+CPT-11)	8 mos	chemo-refractory tumor	Lower abd: incisional scar (1st rec)	None	Surgery, RT	NED/ 140+ mos
11	Otsuka (2017) [2]	53	IIIA1/ Clear cell	Surgery, Chemo (TC)	16 mos	Peritoneal D Axillary, supracl, PA-LNs, Brain	Lower abd: incisional scar	None	RT, immunotherapy	NED/ 93+ mos
12	Otsuka (2017) [2]	54	IIIC/ Serous G2	Surgery, Chemo (TC)	53 mos	Pertioneal D	Lower abd: incisional scar	Perit D, adrenal gland	RT, Chemo (DTX, LPD)	DOD/ 56 mos
13	Traiman (1994) [4]	37	III/ Serous LG	None (exploratory laparotomy)	78 mos	Peritoneal D	Lower abd: incisional scar (1st rec)	None	Chemo (CDDP, epi), RT, Resection	NED/ 72 mos
14	Kohler (1991) [39]	50	IC3/ Mixed G2	Surgery, IP P-32	9 mos	Positive ascites	Lower abd: incisional scar (1st rec)	None	Resection, Chemo (CAP), BCG scarification	NED/ 56 mos
15	Carlson (2002) [40]	77	IIIC/ Serous G3	Surgery, Chemo (platin)	NA	Peritoneal D	Upper right quadrant: port site (1st rec)	None	Resection, Chemo (platin)	NED/ 6+ mos
16	Cormio (2003) [7]	68	IIIC/ Serous G3	Surgery, Chemo (TC)	4 mos	Peritoneal D	Lower abd: incisional scar (1st rec)	Peritoneal D	Surgery, Chemo	AWD/ 4+ mos
17	Cormio (2003) [7]	37	IIIC/ Serous G3	Chemo (TC), Surgery	26 mos	Peritoneal D, Drainage	Drainage scar (1st rec)	Peritoneal D, Liver met, LN met	Chemo	DOD/ 3 mos
18	Woopen (2012) [41]	64	T3cN1/ Serous G2	Surgery, Chemo (TC)	10 wks after 1st-line chemo	Peritoneal D, LN	Lower abd: incisional scar (1st rec)	Peritoneal D, Liver met, LN met	catumaxomab IP	DOD/ NA
19	Cormio (2003) [7]	68	IIIB/ Serous G3	Surgery, Chemo (TC)	33 mos	Port (IP chemo)	Port site (1st rec)	Peritoneal D	Chemo	DOD/ 9 mos
Non-SJN Recurrence after Superficial Lymphadenopathy										
20	Otsuka (2017) [2]	72	IVB/ Adeno G3	Laparomy, Chemo (TC, DTX)	3 mos	Axillary, supracl, inguinal, pelvic LN	Abd (during chemo)	Axillary, supracl, inguinal, pelvic LN	Chemo (primary therapy cont'd)	DOD/ 5 mos
21	Charalampidis (2016) [42]	64	NA/ Serous G2	Surgery, Chemo	10 yrs	Neck LN	Back	Brain	NA	Alive/ 24 mos

22	Kayikciogl (2001) [43]	35	T3c/ Serous G2	Surgery, Chemo (CDDP/PTX)	18 mos	(Axillary LN)	Breast (1st rec)	Axillary LN	Chemo (CDDP/PTX/ anthracyclin)	DOD/ 18 mos
23	Demirci (2010) [8]	43	IIIC/ Serous G3	Surgery, Chemo (Platin)	6 yrs*	Inguinal LN & stump rec	Lower Abd	Inguinal LN, stump	RT	Alive/ 7 mos
24	Kim (2012) [11]	60	T3c/ Serous G3	Surgery, Chemo (Carbo/DTX; TC)	42 mos	Axill,ary supracl LN; Inguinal LN	Groin, Lower extremity	Peritoneal D, Multiple LNs, Muscle	Chemo (Gem/Carbo)	DOD/ 5 mos
25	Yilmaz (2006) [44]	69	T3cM1 (pleura)/ Serous	Chemo (TC) Surgery	31 mos	Pelvic, PA LN (Inguinal LN?)	Lower Abd	LN	Chemo (topo)	DOD/ 4 mos
26	Wiechert (2012) [45]	54	IIC + EmCa/ Endom HG	Surgery, Chemo (TC); Bev	37 mos*	Retroperiton eal LN	Groin, External Genitalia	Retroperitonea l LN, Perit D	RT	DOD/ 10 mos
27	Achimas (2015) [46]	49	IIIC/ Serous G3	Surgery, Chemo (TC); Chemo (topo, gem)	21 mos	Axillary, supra-, infra-diaphragm LN	Chest, Low abd, low extremity	Axillary, supra-, infra-diaphragm LN	Chemo (CPM/Carbo)	DOD/ 1 month
28	Schonmann (2003) [47]	48	IIIC/ Serous G3	Surgery, Chemo (platin/PTX)	42 mos*	Inguinal LN	Lower Abd ~ Upper Thigh	Peritoneal D	Chemo (CDDP)	DOD/ 1 month
Non-SJN Recurrence (Misceraneous)										
29	Otsuka (2017) [2]	49	IIC/ Serous G3	Surgery, Chemo (TC)	40 mos	Peritoneal D Mediastinal, PA LN, Muscle	Hip, Breast	Mediastinal, PA LN, Muscle	Chemo (TC, CPT-11, PLD)	DOD/ 5 mos
30	Yokoyama (1989) [48]	69	II/ Clear cell	Surgery, Chemo (CAF)	9 mos	Pelvic peritoneal D	Chest wall (1st rec)	None	Chemo (CPA, ADR, 5FU); RT; Immuno - stimulant	DOD/ 43 mos
31	Matsui (2002) [49]	64	T2c/ Serous	Surgery, Chemo (PAIfo)	28 mos	Pelvic, PA LN	Scalp (1st rec)	Pelvic, PA LN	RT, Chemo	DOD/ 12 mos
32	Wuntkal (2004) [50]	55	III (FTCa)/ Serous	Surgery, Chemo (Platin)	2 yrs	Peritoneal D	Right thigh, vulva, perineum (1st rec)	Pelvic tumor	Chemo	DOD/ <1–2 month
33	Charalampidis (2016) [42]	34	III/ Serous G2	Surgery, Chemo (PTX; PLD)	2 yrs	Peritoneal D	Chest, Abd, upper/lower exteremities (1st rec)	NA	Chemo	DOD/ 12 mos
34	Lalich (2010) [51]	31	NA/ Endom G2	Surgery	Immedi ately after sugery	NA	Left upper arm (1st rec)	Peritoneal D, Lung, Liver, Brain	Chemo, Surgery	DOD/ 6 mos
35	Nakamura (2012) [52]	63	IV (Perit Ca)/ Serous	Surgery, Chemo (DC)	7 mos	NA	Bilateral thighs (1st rec)	NA	Chemo	AWD/ 9 mos
36	Cokmert (2014) [53]	68	Perit D/ Large cell neuro-endocrine	Palliative RT	2 mos	Peritoneal D Pelvic, PA LN, Lungs, Liver	Leg (progression)	Peritoneal D, Pelvic, PA LN, Lungs, Liver	Chemo (etop + Carbo)	DOD/ 5 mos
37	Patsner (1988) [3]	70	N1/ Adeno G3	Surgery, Chemo (CDDP + CPM)	NA	PA LN	Abd, Groin, Thigh, Perineum	Abd, Groin, Thigh, Perineum	Chemo (CDDP + CPM)	DOD/ <1 mo

Skin recurrences after laparoscopic surgery and paracenteses are excluded. Endom, endometrioid; LG, low-grade; EmCa, endometrial cancer; FTCa, fallopian tube cancer; Perit Ca, peritoneal cancer. Peritoneal D, peritoneal dissemination; LN lymph node; PA, para-aortic; abd, abdomen; supracl, supraclavicular; NA, not available. TC, paclitaxel (PTX)/carboplatin (Carbo); CAP, cyclophosfamide (CPM)/Adriamycin (ADR)/cisplatin (CDDP); cap, capecitabine; oxa, oxaliplatin; MMC, mitomycin C; CPT-11, irinotecan; DTX, docetaxel; topo, topotecan; gem, gemcitabine; CAF, CPM/ADR/5-FU; Ifo, ifosfamide; PLD, pegylatd liposomal doxorubicin; DC, DTX/Carbo; epi, epirubicin; etop, etoposide; RT, radiation therapy; IP, intraperitoneal; AWD, alive with disease; DOD, dead of disease; NED, no evidence of disease.

Table S4. Port-site recurrence of borderline ovarian tumor.

No	Author (Year)	Age	FIGO Stage	Histotype	Type of Surgery	Risk Factors	Time Interval	Treatment	Status/ Survival
1	Hsiu (1986) [54]	30	IIB	Serous	Biopsy of OvT	Papillary excresences on both ovaries, Drainage, Ascites, Peritoneal implants	3 wks	Surgery, Chemo (CAP)	NED/ 9 mos
2	Hsiu (1986) [54]	28	III	Serous	Biopsy of OvT	Papillary excresences on both ovaries, Ascites, Peritoneal & omental implants	3 wks	Surgery, ip P32, Chmeo (Mel, CAP)	NED/ >6 yrs
3	Gleeson (1993) [55]	31	IIB	Serous	LSO, Biopsy of rt. OvT	Papillary excresences on both ovaries, Pertitoneal implants	2 wks	Surgery, Chemo (CP)	Alive/ NA
4	Shepherd (1994) [56]	23	I	Serous	Cystectomy	None	6 wks	Surgery	NED/ 24 mos
5	Morice (2004) [57]	28	I	Serous	Cystectomy	Recurrence of serous borderline lt. OvT, excrescences on rt. ovary	11 mos	Surgery	NED/ 11 mos
6	Morice (2004) [57]	28	II	Serous	Biopsy of OvT	Bilataral OvTs, Peritoneal implants	3 mos	Surgery	NED/ 23 mos
7	Morice (2004) [57]	55	I	Mucinous	RSO	None	1 mo	Surgery	NED/ 51 mos
8	Furukawa (2014) [58]	50	IA	Mucinous*	LSO	None	26 mos	Surgery, Chemo (TC)	Alive/ NA

* Invasive recurrence of mucinous ovarian tumor at a port site. OvT, ovarian tumor; LSO, left salpingo-oophorectomy; RSO, right SO; NED, no evidence of disease; NA, not available. CAP, cyclophosfamide (CPM)/Adriamycin (ADR)/cisplatin (CDDP); Mel, melphalan; CP, CPM/CDDP; TC, paclitaxel/carboplatin

© 2019 by the authors. Licensee MDPI, Basel, Switzerland. This article is an open access article distributed under the terms and conditions of the Creative Commons Attribution (CC BY) license (http://creativecommons.org/licenses/by/4.0/).
